# Antibacterial Potential of an Antimicrobial Agent Inspired by Peroxidase-Catalyzed Systems

**DOI:** 10.3389/fmicb.2017.00680

**Published:** 2017-05-02

**Authors:** Lilit Tonoyan, Gerard T. A. Fleming, Paul H. Mc Cay, Ruairi Friel, Vincent O'Flaherty

**Affiliations:** ^1^Microbiology, School of Natural Sciences and Ryan Institute, National University of Ireland GalwayGalway, Ireland; ^2^Westway Health Ltd., Business Innovation Centre, National University of Ireland GalwayGalway, Ireland

**Keywords:** iodo-thiocyanate complex (ITC), biocide, iodine, *in vitro* susceptibility, bactericidal

## Abstract

Antibiotic resistance is an increasingly serious threat to global health. Consequently, the development of non-antibiotic based therapies and disinfectants, which avoid induction of resistance, or cross-resistance, is of high priority. We report the synthesis of a biocidal complex, which is produced by the reaction between ionic oxidizable salts—iodide and thiocyanate—in the presence of hydrogen peroxide as an oxidation source. The reaction generates bactericidal reactive oxygen and iodine species. In this study, we report that the iodo-thiocyanate complex (ITC) is an effective bactericidal agent with activity against planktonic and biofilm cells of Gram-negative (*Escherichia coli* and *Pseudomonas aeruginosa*) and Gram-positive (*Staphylococcus aureus* and methicillin-resistant *S. aureus*) bacteria. The minimum bactericidal concentrations and the minimum biofilm eradication concentrations of the biocidal composite were in the range of 7.8–31.3 and 31.3–250 μg ml^−1^, respectively. As a result, the complex was capable to cause a rapid cell death of planktonic test cultures at between 0.5 and 2 h, and complete eradication of dual and mono-species biofilms between 30 s and 10 min. Furthermore, the test bacteria, including a MRSA strain, exposed to the cocktail failed to develop resistance after serial passages. The antimicrobial activity of the ITC appears to derive from the combinational effect of the powerful species capable of oxidizing the essential biomolecules of bacteria. The use of this composition may provide an effective and efficient method for killing potential pathogens, as well as for disinfecting and removing biofilm contamination.

## Introduction

The phenomenon of antibiotic resistance is expanding and the threat regarding our future ability to combat infection is increasing (WHO, 2014). Thus, key challenges for society and for researchers are to address microbial drug resistance and to develop non-antibiotic therapies.

The antimicrobial properties of naturally occurring peroxidase systems are well-known. Human exocrine secretions such as milk, saliva, tears, seminal, vaginal, and gastrointestinal fluids; as well as human phagocytic cells, such as neutrophils, monocytes and eosinophils, contain peroxidase enzymes, which comprise part of the innate host defense system (Ihalin et al., 2006; Davies et al., 2008). Peroxidases alone have no antibacterial effect. However, a peroxidase exerts an antimicrobial effect indirectly by catalyzing the transformation of a substrate with low antimicrobial properties into one with high antimicrobial effects (Klebanoff, 1967). The complete antimicrobial peroxidase system requires three components: a particular peroxidase enzyme, hydrogen peroxide (H_2_O_2_), and an oxidizable substrate such as a halide or a pseudohalide (Ihalin et al., 1998). Peroxidase-catalyzed oxidation of (pseudo)halides yields reactive agents which oxidize microorganisms, damaging essential structural and functional components and causing inhibition of microbial metabolism and growth (Thomas and Fishman, 1986).

Different peroxidases preferably oxidize different (pseudo)halides, generating distinct antimicrobial species. For example, myeloperoxidase (MPO) of neutrophils employs chloride as a substrate and forms hypochlorous acid as the main product (Klebanoff, 2005). Lactoperoxidase (LPO) of milk and salivary peroxidase (SPO) of saliva readily oxidize thiocyanate (SCN^−^) and generate hypothiocyanous acid or its conjugate base hypothiocyanite (OSCN^−^), the latest being predominant in most physiological fluids (Chandler and Day, 2012). Iodide (I^−^) can also be oxidized by MPO, LPO, and SPO and it is the most readily oxidizable of all halides *in vitro*. The peroxidase-catalyzed oxidation of I^−^ yields molecular iodine (I_2_) and, depending on I^−^ concentration and pH, hypoiodous acid and hypoiodite (OI^−^), or else, other iodine species may be present (Kussendrager and van Hooijdonk, 2000).

The antimicrobial properties of peroxidase systems arouse much interest toward their *in vitro* and *in vivo* applications. The use of peroxidase systems as antimicrobial agents has, however, been somewhat limited, because the cost of the enzyme purification is often higher than traditional preservatives, and sourcing large quantities of enzymes can be a problem. Large-scale purification of peroxidases from human leukocytes (MPO) or from human milk (LPO) and human saliva (SPO) is relatively difficult and expensive. These enzymes have been purified but only for research objectives. LPO purified from bovine milk has been extensively used for research and commercially as it is readily available. LPO systems have broad spectrum of actual and potential applications as natural biopreservatives in oral healthcare, milk industry, food, feed specialties, cosmetics, and related products, which were extensively reviewed elsewhere (de Wit and van Hooydonk, 1996; Kussendrager and van Hooijdonk, 2000; Seifu et al., 2005; Ciccognani, 2006).

We have developed an iodo-thiocyanate complex (ITC), which has similarities to peroxidase systems in that it is based on halide oxidation, yet has two differences—it is comprised of two substrates, iodide and thiocyanate, and does not employ a peroxidase enzyme. Indeed, though the literature indicates that the peroxidase enzyme is required to catalyze the reaction between I^−^, SCN^−^ and low concentrations of H_2_O_2_ in order to exert antimicrobial activity, results from our *in vitro* research indicates that H_2_O_2_/I^−^/SCN^−^ combination rapidly generates antimicrobial species, even at low concentrations, without the presence of any peroxidase enzyme. Antimicrobial combinations represent a therapeutic option in the treatment of infections. In general, the combination therapy is used to avoid the development of antimicrobial resistance (as the use of two or more antimicrobials with different targets decreases the possibility that an organism will possess the features necessary for survival) and to enhance the efficacy of the individual antimicrobials through synergistic interactions. Whether a combinational approach could be adopted to increase the antimicrobial potency of a peroxidase-like system by involvement of two halides, was examined in this study. In addition, the study evaluated the antibacterial properties of the new antimicrobial combination, elucidated potential reactive species involved in its antimicrobial performance and illustrated its effect on bacterial cell ultrastructure.

## Materials and methods

### Antimicrobial agents and preparation

All the materials were purchased from Sigma-Aldrich unless otherwise stated. The antimicrobial agents evaluated in this study included: H_2_O_2_ and its combinations with potassium iodide (H_2_O_2_/KI), with potassium thiocyanate (H_2_O_2_/KSCN) and with both (H_2_O_2_/KI/KSCN, named ITC). The stock solutions of H_2_O_2_/KI and H_2_O_2_/KSCN were prepared by combining H_2_O_2_ and KI or KSCN, respectively, at a ratio of 1:1 (v/v) to reach 1% final concentrations for each agent. These solutions were considered as 1%, according to the concentration of H_2_O_2_ present in the mixtures, since KI and KSCN alone do not possess antimicrobial properties. ITC stock solution was a mixture of H_2_O_2_/KI/KSCN at a 1:1:1 ratio (v/v/v) with 1% final concentration of each component (this solution was considered as 1% ITC). Due to high reactivity the stock solution of H_2_O_2_/KI was prepared freshly before use and used within 15 min, whereas, H_2_O_2_/KSCN and ITC were stable for a long time period (over 6 months) and were stored at 4°C. Stock solutions (1%) of H_2_O_2_ and three test mixtures were diluted to appropriate working concentrations using sterile deionized water (dH_2_O) or nutrient broth of choice. Antibiotics polymyxin B (PMB), levofloxacin (LVX); antiseptics povidone iodine (PVP-I) and Lugol's iodine (Lugol) were also used in this study. Their working dilutions were prepared using sterile dH_2_O or LB (for LVX).

### Bacterial strains and inoculation

Bacteria used in this study included: *Escherichia coli* ATCC 25922, *Pseudomonas aeruginosa* NCIMB 10421, *Staphylococcus aureus* DSM 15676, *S. aureus* BH1CC (methicillin-resistant *S. aureus* (MRSA) clinical isolate), *Streptococcus uberis* (mastitis isolate). All bacterial strains were cultured in lysogeny broth (LB) and Lennox agar (LA), and incubated aerobically at 37°C throughout the study. Trypticase soy agar (TSA) supplemented with 5% sheep blood (Fannin, Dublin, Ireland) was used for the differential characterization of Streptococcus and Staphylococcus species.

### Antibacterial screening by disc diffusion method

For initial comparison of the antimicrobial activity of H_2_O_2_ with its three different combinations, the susceptibility screening of four test strains (*E. coli, P. aeruginosa, S. aureus*, and MRSA) to the antimicrobial combinations was performed using standard technique (CLSI, 2012b). Aliquots (100 μl) of freshly prepared phosphate-buffered saline (PBS) suspension of each bacterial strain at optical density of OD_625_ = 0.1, corresponding to 10^8^ colony-forming units per ml (cfu ml^−1^), was used to lawn the LA plate. The Whatman filter discs (6 mm) were impregnated with 10 μg of antimicrobial mixtures (estimated by the amount of H_2_O_2_ only) and incubated for 24 h at 37°C. Antimicrobial activity was evaluated by measuring the diameter of the growth inhibition zones (ZOI). Likewise, for concentration-dependent effect of ITC, discs were charged with various doses of ITC (10, 20, 40, and 80 μg disc^−1^) and ZOI were measured. Each strain, each antimicrobial and each concentration of ITC were tested in duplicates on three separate occasions.

### Minimum inhibitory concentrations (MIC) and minimum bactericidal concentrations (MBC) by broth microdilution method

The MICs and MBCs of H_2_O_2_, H_2_O_2_/KI, H_2_O_2_/KSCN and ITC were determined against *E. coli, P. aeruginosa, S. aureus*, and MRSA test strains using a broth microdilution method according to standard guidelines (CLSI, 1999, 2012a). Bacteria at 5 × 10^5^ cfu ml^−1^ final density were seeded in 96-well plates and challenged with the specified compounds at serial 2-fold dilutions. The growth of the strains was monitored in microtiter plate reader (Tecan GENios, Salzburg, Austria). OD_595_ measurements were recorded at 15 min intervals over 24 h at 37°C. The MIC was defined as the lowest concentration of antimicrobial agent preventing the appearance of turbidity. MBCs of the antimicrobials were determined by sub-culturing the content of the no growth wells from the above MIC test onto solid media. The MBC values were defined as the lowest concentrations which produced no colonies on the agar plates. The experiments were performed on five occasions. MICs and MBCs of the test compounds from independent experiments varied in the quintuple, but only by one concentration higher or lower in the dilution series. Accordingly, the modes of each dataset were reported as MIC or MBC. The antimicrobial was considered to exhibit bactericidal activity when the MBC/MIC ratio was ≤4 (Pankey and Sabath, 2004).

### Microbial killing rates by time-kill assay

Time-dependent killing of H_2_O_2_ and its three derivatives were identified against *E. coli, P. aeruginosa, S. aureus*, and MRSA strains using standard technique (Moody and Knapp, 2004). Test cultures at 5 × 10^5^ cfu ml^−1^ density were exposed to all antimicrobial combinations at the concentration of 31.3 μg ml^−1^ in 5 ml LB. In order to have comparative results, for all the test strains and for all the test antimicrobials 31.3 μg ml^−1^ concentration was chosen as it was the most frequently occurring MBC value (Table 1). Inoculum in broth without any antimicrobial was considered as a control. After the inoculation all the suspensions were incubated at 37°C under continuous shaking conditions. At predetermined time points (0, 0.5, 1, 2, 4, 8, and 24 h), aliquots (25 μl) were aseptically removed, serially diluted in PBS and plated on LA plates. The plates were incubated at 37°C, and cell survival was determined by colony counts. The determinations were done in duplicates for two occasions.

**Table 1:** **Minimum inhibitory (MIC) and minimum bactericidal concentrations (MBC) (expressed as μg ml^**−1**^) of test antimicrobial combinations against representative strains determined by broth microdilution method**.

**Bacterial strain**	**H**_**2**_**O**_**2**_		**H**_**2**_**O**_**2**_**/KI**		**H**_**2**_**O**_**2**_**/KSCN**		**ITC**	
	**MIC**	**MBC**	**MIC**	**MBC**	**MIC**	**MBC**	**MIC**	**MBC**
*E. coli* ATCC 25922	31.3	31.3	31.3	31.3	31.3	31.3	15.6	15.6
*P. aeruginosa* NCIMB 10421	62.5	125	62.5	125	62.5	125	31.3	31.3
*S. aureus* DSM 15676	7.8	7.8	7.8	7.8	7.8	7.8	7.8	7.8
*S. aureus* BH1CC (MRSA)	15.6	31.3	15.6	31.3	15.6	31.3	15.6	31.3

*Data are shown as the mode of n = 5*.

### Biofilm growth on modified robbins device (MRD) and determination of minimum biofilm eradication concentrations (MBEC) by viable cell count

Susceptibility of H_2_O_2_/KI/KSCN combination (ITC) was determined for mono- and dual-species biofilm bacteria. Mono-species biofilm modes of *E. coli* ATCC 25922, *P. aeruginosa* NCIMB 10421, *S. aureus* DSM 15676, and MRSA BH1CC strains, and dual-species biofilm of *S. aureus* DSM 15676 and *S. uberis* were grown as a batch culture coupled with MRD, wherein, the bacterial cells were allowed to attach and proliferate on the surface of polyurethane coupons with their “face” exposed to the recirculating flow over time (Supplementary Figure 1; Kharazmi et al., 1999). In brief, MRD is an acrylic multiport sampling chamber containing 12 ports in a linear array (Tyler Research Corporation, Edmonton, Canada). Each port accepts a press-fit plug holding a polyurethane coupon (surface area 50 mm^2^). Coupons were placed on top of the flow, without disturbing flow characteristics. The MRD was connected to peristaltic pump (Watson-Marlow 205S, Falmouth, UK) and growth media reservoir by silicone tubing. The device was disinfected with 1% (w/v) Virkon (Anachem Ltd, Leicester, UK), the plugs and the coupons were exposed to 100% ethanol, the media bottle and the tubing were autoclaved before and after each run. To establish the mono-species biofilms, the media reservoir containing 300 ml LB was seeded with test bacteria at 10^6^ cfu ml^−1^ density. For co-culturing *S. aureus* and *S. uberis*, 300 ml LB reservoir was seeded with two strains at final density of 10^6^ cfu ml^−1^ each. Broth media, containing given bacteria, was pumped through the device at a rate of 0.1 h^−1^ under 37°C conditions. After 24 h individual coupons protruding into the flow channel were taken out and prewashed with PBS (removing any unbound cells), exposed to ITC treatment at serial 2-fold dilutions upon 10 min for mono-species biofilms and 30 s for dual-species biofilm and washed again (washing away the antimicrobial agent). The coupons were then transferred to fresh 1 ml PBS in a 2 ml mini-centrifuge tubes. The biofilm cells were removed from the coupons by vortexing 1 min and sonicating in a sonication bath for 10 min (Branson Ultrasonics Corporation, Danbury, USA). Viable cell count on LA plates was used to establish bacterial numbers of mono-species biofilms post-treatment. For differential selection of Staphylococcus and Streptococcus species, aliquots were plated on TSA + blood plates and were enumerated according to the type of hemolysis. For single-species biofilms the results were expressed as log_10_ cfu ml^−1^ values, while for mixed biofilms results were relative proportion of each bacterial component expressed at the % of the total population in mixed biofilms. At each concentration of the antimicrobial treatment cfu values were counted from four coupons. The MBEC of ITC was determined as the minimum concentration that prevents the growth in the recovery medium used to collect biofilm cells (0 cfu coupon^−1^ on plate counts; Macia et al., 2014).

### Potential for resistance using sequential passage in the presence of the antimicrobial

The persistence of antimicrobial susceptibility in experimental populations of bacteria was tested over 20 passages by broth microdilution method in accordance with the procedure described elsewhere (Friedman et al., 2006; D'Lima et al., 2012). Multipassage resistance studies using H_2_O_2_, ITC, and LVX were performed for *E. coli, P. aeruginosa, S. aureus*, and MRSA strains. At day 1, LB containing twelve 2-fold dilutions of each of antimicrobial drugs—H_2_O_2_, ITC (starting at 500 μg ml^−1^) and LVX (starting at 8 μg ml^−1^), and LB without drug (growth control, GC) were seeded with bacteria at final density of 5 × 10^5^ cfu ml^−1^ in 96-well plates. Cultures were incubated 24 h in 37°C incubator. For each subsequent daily passage, for each test antimicrobial and strain, aliquots were taken from the wells with concentrations one to two dilutions below the MIC (that matched the turbidity of a GC well) and were used to inoculate the dilution series for the next day, so that the bacteria were again seeded at 5 × 10^5^ cfu ml^−1^. H_2_O_2_, ITC, and LVX were added to the wells containing these bacterial suspensions at 2-fold dilutions. These cultures represented the exposed groups. In a same manner, bacterial culture from the GC well (the well cultured without antimicrobial agent from the previous passage) was diluted to 5 × 10^5^ cfu ml^−1^ into fresh media, dispersed into the wells and H_2_O_2_, ITC, and LVX were added at the same 2-fold dilutions, representing the unexposed groups. The process was continued for 20 passages and the MIC values for exposed and unexposed groups were recorded. The relative MIC was calculated for each passage from the ratio of MIC obtained from an exposed culture to that obtained from an unexposed culture.

### Identification of the reactive species

A number of analytical methods were used to identify and quantify the chemical species generated within the antimicrobial mixtures used in this study. Solutions (1%—according to the concentration of H_2_O_2_ present) of H_2_O_2_/KSCN and H_2_O_2_/KI/KSCN were prepared the day before the measurements and their peroxide, hypoiodite/hypothiocyanite, hydroxyl radical, and iodine contents were measured. H_2_O_2_/KI was prepared freshly for each measurement, due to its weak stability and rapid loss of antimicrobial activity. Iodine content was also measured in 1% solutions of PVP-I and Lugol's iodine. All the measurements were performed on duplicate solutions for each test antimicrobial.

### Detection of hydrogen peroxide

Quantofix Peroxide 100 semi-quantitative test strips were used to detect the concentrations of unreacted H_2_O_2_ in the test antimicrobial solutions. The tests were carried out in accordance with manufacturer's instructions.

### Measurement of hydroxyl radical (·OH)

The concentrations of ·OH in 1% solutions of test antimicrobials were detected using a dimethyl sulfoxide (DMSO) as a molecular probe (Babbs and Steiner, 1990). Chemically, DMSO traps ·OH and is oxidized to a single, stable, non-radical product, methanesulfinic acid (MSA). The measurement of MSA accumulation in DMSO pretreated systems provides a potential mean to capture and count the ·OH generated therein. Further, MSA can be assayed colorimetrically based on the reaction with diazonium salts, particularly, fast blue BB salt. The product is a colored diazosulfone, which can be selectively extracted into an organic solvent and measured spectrophotometrically at 425 nm. The color reaction was carried out according to the assay developed by Babbs and Steiner (1990). The concentration of sulfinic acid was calculated from an MSA standard curve.

### Measurement of hypothiocyanite/hypoiodite

Concentrations of OSCN^−^/OI^−^ were measured based on the oxidation of sulfhydryl compound 5-thio-2-nitrobenzonic acid (TNB) which absorbs at 412 nm (Aune and Thomas, 1977; Bosch et al., 2000; Cegolon et al., 2014). Firstly, DTNB [5,5′-dithiobis(2-nitrobenzoic acid)] is reduced to TNB with sodium borohydride (NaBH_4_). When mixing yellow-colored TNB with OSCN^−^/OI^−^, these will reoxidize TNB to colorless DTNB. Each mole of DTNB will yield 2 moles of TNB, and each 2 mole of TNB are reoxidized to DTNB by 1 mole of OSCN^−^ (or OI^−^ or OSCN^−^/OI^−^). Particularly, the reactive solution was prepared by mixing 40 mg of DTNB with 20 mg of NaBH_4_ and brought to 100 mL with 0.5 M Tris–HCl buffer pH 7. The absorbance of the samples (0.25 ml reactive in 2.65 ml dH_2_O and 0.1 ml sample antimicrobial solution) was measured and compared to the absorbance of reference (0.25 ml reactive in 2.75 ml dH_2_O). OSCN^−^ or OI^−^, or OSCN^−^/OI^−^ concentration was calculated by employing the following formula:

$$\begin{array}{l} \left[\text{OSC}{\text{N}}^{-}/\text{O}{\text{I}}^{-}\right]\left(\text{mM}\right)\;=\;\frac{\left(\Delta \text{OD}\right)\;\times \;3\;\times \;1,000}{13,600\;\times \;2\;\times \;0.1} \end{array}$$

where, ΔOD = (OD_reference_) − (OD_OSCN_−_/OI_− _solution_); 3 = total volume; 0.1 = sample volume; 13,600 M^−1^ cm^−1^ = molar extinction coefficient of DTNB; 2 = correction factor for the stoichiometric reaction (2 moles TNB reacts with 1 mole OSCN^−^/OI^−^ to produce 1 mole DTNB); 1,000 = M bring to mM.

### Measurement of iodine

I_2_ content in all test antimicrobial aqueous solutions was measured by the iodometric titration method as described by Sullivan et al. (1995) which is based on titration with sodium thiosulfate (Na_2_S_2_O_3_). Free iodine is consumed by Na_2_S_2_O_3_ and converted to iodide according to the equation:

$$\begin{array}{l} {\text{I}}_{2}\;+\;2\;{\text{S}}_{2}{\text{O}}_{3}^{2-}\;\to \;2\;{\text{I}}^{-}\;+\;{\text{S}}_{4}{\text{O}}_{6}^{2-} \end{array}$$

Briefly, 500 μl of 1% antimicrobial mixtures were titrated with a 5 mM Na_2_S_2_O_3_ standard solution until colorless solutions were obtained. Near the end-point, a 100 μl of 1% starch (BDH Laboratory Supplies, Poole, England) solution was added as an external indicator of this reaction, which reacts with I_2_ to produce a blue color and to enhance the color change, and titration was continued further. The amount of iodine present is proportional to the amount of thiosulfate used and was calculated according the formula:

$$\begin{array}{l} \text{C }\left({\text{I}}_{2}\right)=\;\frac{\text{C }\left({\text{Na}}_{2}{\text{S}}_{2}{\text{O}}_{3}\right)\;\times \text{ V }\left({\text{Na}}_{2}{\text{S}}_{2}{\text{O}}_{3}\right)}{2\;\times \text{ V }\left({\text{I}}_{2}\right)} \end{array}$$

where, V (I_2_) is the initial volume of the test solution, V (Na_2_S_2_O_3_) is the volume of thiosulfate added, C (Na_2_S_2_O_3_) is the concentration of thiosulfate.

### Detection of iodine species

Iodine species (I^−^, I_2_, and ${\text{I}}_{3}^{-}$; tri-iodide) in 1% aqueous solutions of H_2_O_2_/KI and H_2_O_2_/KI/KSCN combinations were also qualitatively observed using UV-Vis spectrometry (Gazda et al., 2004) and compared to spectra of commercially available iodine containing mixtures—PVP-I and Lugol's iodine. UV-Vis absorption spectra over a range of 190–700 nm were obtained at room temperature on a Varian Cary 50 Scan UV-visible spectrophotometer, using a quartz cuvette of 1 cm optical length. Where there were overlapping bands or oversaturation, 1% stock solutions of different antimicrobials were diluted at various dilution factors (1/2, 5, 10, 100, 200, 500, 1,000).

### Transmission electron microscopy (TEM) to detect structural changes in bacteria

TEM was used to assess the effect of ITC on bacterial cell ultrastructure. *E. coli* cells were grown to exponential phase and 2.5 × 10^8^ cfu ml^−1^ were treated with ITC at 30 and 300 μg ml^−1^, and H_2_O_2_ at 300 μg ml^−1^ for 2 h at 37°C. Before proceeding to TEM sample preparation, aliquots from the treated and untreated cultures were aseptically removed and plated on agar plates for viable cell counts. Next, the cell pellets were washed with PBS and fixed with 2.5% glutaraldehyde (Serva, Heidelberg, Germany) in PBS for overnight. Afterwards, the pellets were washed and post-fixed with buffered (PBS) 1% osmium tetroxide for 2 h. Samples were then dehydrated in a graded series of ethanol (50, 75, 90, and 100%), followed by 1 h incubation with propylene oxide. They were further infiltrated with epoxy resin (TAAB Laboratories Equipment Ltd, Aldermaston, UK) according to manufacturer's protocol. Samples were polymerized at 65°C for 72 h. Ultrathin sections were cut at 90 nm (Reichert-Jung Ultramicrotome equipped with diamond knife) and deposited on 200-mesh copper grids. Specimens were post-stained with 0.5% uranyl acetate and 3% lead citrate with automated contrasting apparatus (Leica EM AC20). Sections were observed in a transmission electron microscope (Hitachi H7000) at 75 kV under 10,000× and 20,000× magnifications.

### Agarose gel electrophoresis to detect bacterial DNA damage

The potential of ITC as an agent that would induce bacterial DNA breakage (DNA smear) was examined by agarose gel electrophoresis (Yoon et al., 2002; Kasibhatla et al., 2006). Briefly, exponential phase *E. coli* cells were treated with ITC and H_2_O_2_ at 30 and 300 μg ml^−1^ for 30 min, and genomic DNA of each sample was extracted using the QIAamp DNA Mini Kit (Qiagen GmbH, Hilden, Germany) according to the manufacturer's instructions. gDNA samples were analyzed by electrophoresis on a 2% agarose gel containing ethidium bromide (10 μg ml^−1^; Promega Corporation, Medison, USA). gDNA samples were electrophoresed at 40 V until the tracking dye (Blue/Orange 6x Loading Dye; Promega, Madison, USA) front reached the bottom of the gel and then visualized under the UV illuminator (Azure c200, Azure Biosystems).

### Live/dead staining and fluorescent microscopy to evaluate bacterial outer membrane damage

The impact of ITC on membrane integrity of planktonic and biofilm *E. coli* cells was observed by LIVE/DEAD BacLight staining (Molecular Probes Inc, Eugene, USA), which is widely used assay to demonstrate membrane damage (Ercan et al., 2014). Planktonic cells of *E. coli* in exponential phase were treated with 125 μg ml^−1^ concentration of ITC for 1 h, washed and stained according to manufacturer's indications. As red-labeled staining control polymyxin B at 1 μg ml^−1^ (2 × MIC) concentration was used, as it acts on bacterial membranes, making it more permeable (Storm et al., 1977). Prior to staining, aliquots were taken from untreated, ITC and PMB-treated planktonic cultures for standard plate counts. For assays with biofilms, *E. coli* cultures were grown on glass coverslips over 24 h than washed to remove media and planktonic cells. Established biofilms were than treated with three different concentrations (7.8, 31.3, and 125 μg ml^−1^) of ITC mixture for 10 min, washed and stained accordingly. Bacteria were observed under Nikon Eclipse E600 microscope with a 100 × oil immersion objective lens, equipped with a QICAM Fast 1394 camera. Live/dead composite fluorescent images were acquired using QCapture Pro 5.1 software.

## Results

### Antibacterial profile of new antimicrobial mixtures

**Figure 1:**
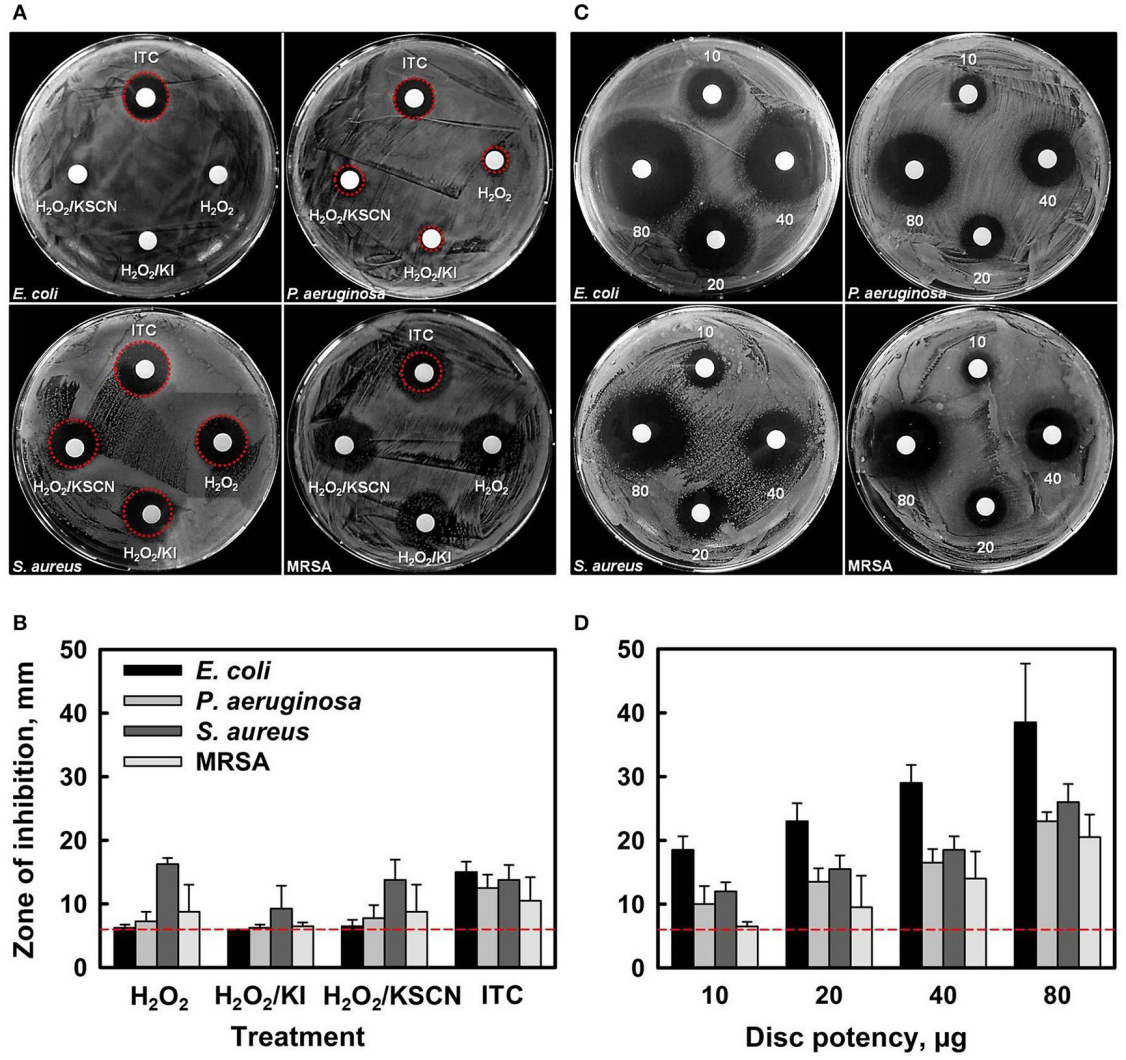
**Antibacterial activity of H_**2**_O_**2**_, H_**2**_O_**2**_/KI, H_**2**_O_**2**_/KSCN, and ITC against ***E. coli*** ATCC 25922, ***P. aeruginosa*** NCIMB 10421, ***S. aureus*** DSM 15675 and ***S. aureus*** BH1CC (MRSA) strains (A,B)**. Representative sample agar plates showing the zones of inhibition formed by antimicrobials (10 μg of each antimicrobial combination was applied) **(A)**. Mean diameter of the ZOI (in mm, including the 6 mm diameter of the disc) of different antimicrobials recorded in three independent assays performed in duplicates, error bars indicate +SD **(B)**. Dose-dependent antimicrobial activity of ITC against the test strains **(C,D)**. Representative sample agar plates showing the ZOIs around the discs impregnated with different amounts of ITC (10, 20, 40, and 80 μg disc^−1^) **(C)**. Mean diameter of the ZOI of different amounts of ITC recorded in three independent assays performed in duplicates (+SD) **(D)**. Dotted lines represent level of no activity.

Initial antibacterial screening of H_2_O_2_ and its combinations with KI, or KSCN, or both KI and KSCN, toward two Gram^−^ (*E. coli* and *P. aeruginosa*) and two Gram^+^ (*S. aureus* and MRSA) bacteria was carried out by disc diffusion assays (Figures 1A,B). Antimicrobial susceptibility discs, loaded with 10 μg of each antimicrobial combination (estimated according to the amount of H_2_O_2_ only) showed that ITC had antimicrobial activity toward all the test organisms. By contrast, H_2_O_2_, H_2_O_2_/KI, and H_2_O_2_/KSCN did not cause inhibition of *E. coli* growth and induced only very slight inhibition of *P. aeruginosa*. The antimicrobial combinations under test had similar efficacies for *S. aureus* and MRSA strains. The preliminary screening by disk diffusion demonstrated that combination of two substrates has a synergistic antimicrobial effect. Subsequently, the dose-dependent inhibition effect of ITC was examined, demonstrating that the ZOIs increased with increasing concentration of ITC for all four test strains (Figures 1C,D).

MIC and MBC values of tested antimicrobial compounds are listed in Table 1. ITC showed moderate to high activity toward all test organisms with MIC and MBC values ranging 7.8–31.3 μg ml^−1^. In comparison to the other test antimicrobials, MICs and MBCs were lower for ITC, against *E. coli* and *P. aeruginosa*, and were similar against *S. aureus* and MRSA. This outcome is in agreement with disk diffusion results presented in Figure 1. MBC/MIC ratios indicated that all antimicrobials exerted a bactericidal effect on the studied strains.

**Figure 2:**
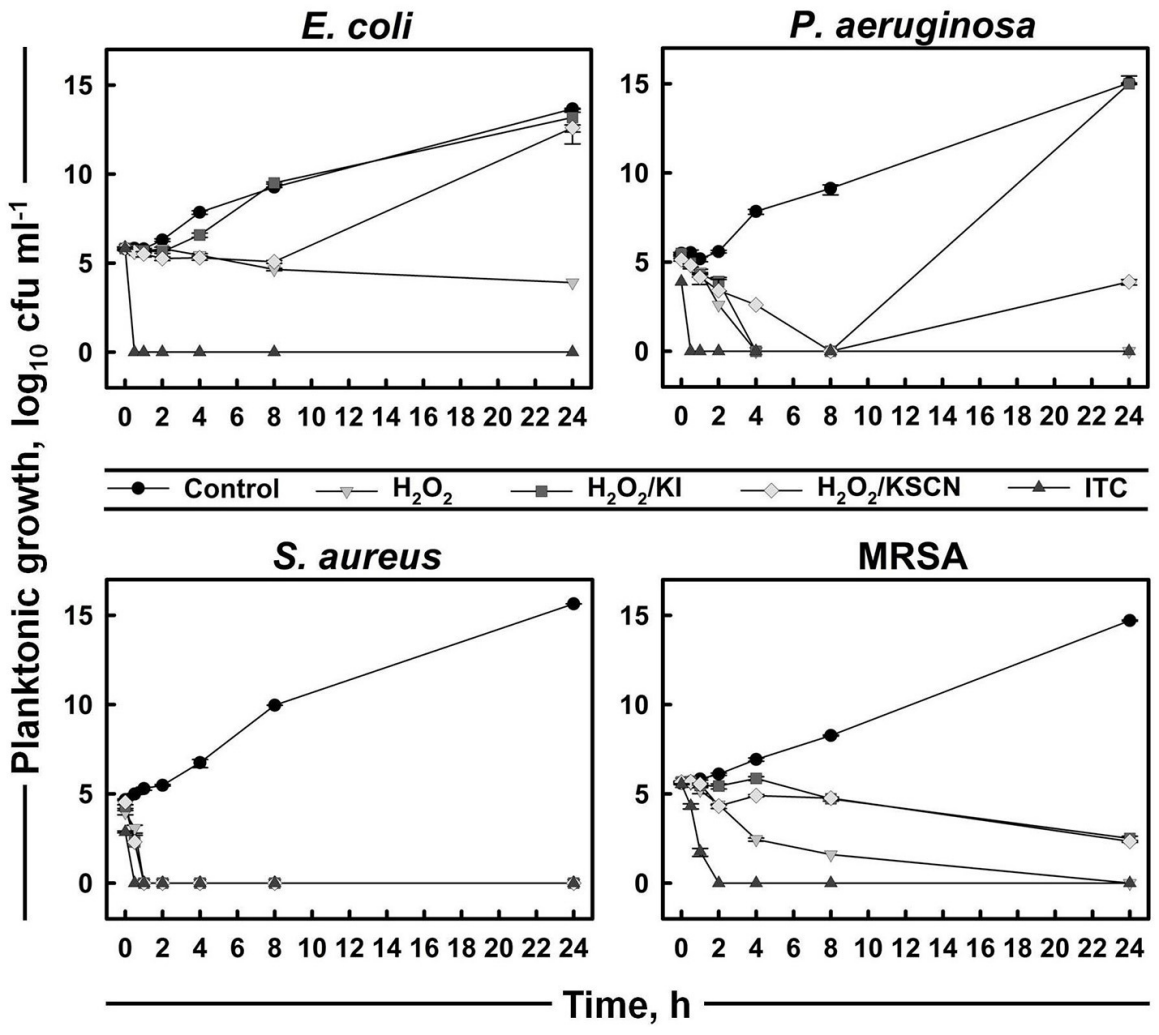
**Time-kill curves of representative strains treated with H_**2**_O_**2**_, H_**2**_O_**2**_/KI, H_**2**_O_**2**_/KSCN, and ITC**. Concentration of each antimicrobial mixture is 31.3 μg ml^−1^. Mean values of duplicate cfu ml^−1^ measurements (± SD) are plotted.

A time-kill assay was used to further evaluate the bactericidal activities of the antimicrobials (Figure 2). The time-course of bacterial viability was determined after bacterial four test strains were treated with H_2_O_2_ and its derivatives at the concentration of 31.3 μg ml^−1^ (which was the most frequent MBC value within the strains and the antimicrobials; Table 1). The results showed that *E. coli, P. aeruginosa*, and *S. aureus* were killed within 30 min after the addition of ITC. Moreover, *P. aeruginosa* and *S. aureus* exposed to ITC showed reduced bacterial load compared with the unexposed control, even at initial sampling time (T_0_), indicating that killing of this strains by ITC occurred immediately. Killing of MRSA was achieved after 2 h of exposure to ITC. *S. aureus* was also sensitive toward the rest of the test antimicrobials and its complete killing was observed within 30 min of exposure. H_2_O_2_ at the tested concentration (31.3 μg ml^−1^) could not inhibit *E. coli*, or required longer time to eliminate *P. aeruginosa* and MRSA strains, within 4 and 24 h, respectively. H_2_O_2_/KI and H_2_O_2_/KSCN combinations could not arrest the growth of *E. coli, P. aeruginosa*, and MRSA (Figure 2).

**Figure 3:**
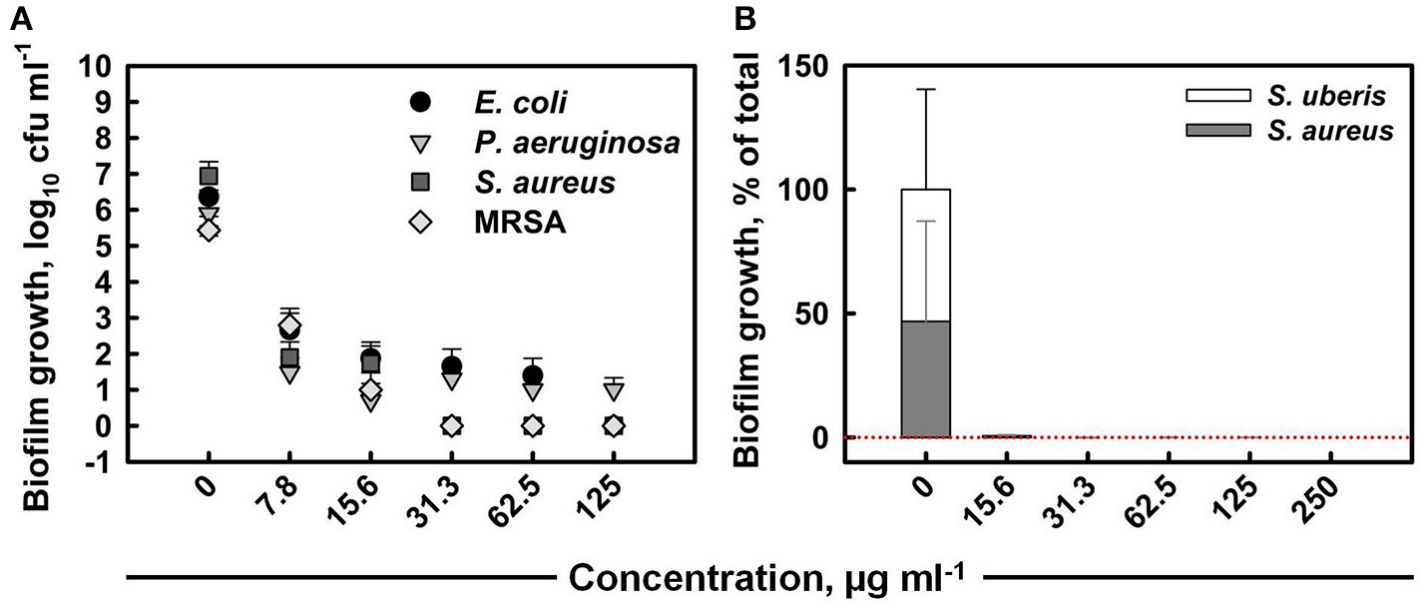
**Anti-biofilm activity of ITC toward single-species biofilms of ***E. coli***, ***P. aeruginosa***, ***S. aureus***, and MRSA strains at different concentrations upon 10 min interaction (A)**. Mean values of tetrad coupon log_10_ cfu ml^−1^ measurements (± SD) are plotted. Anti-biofilm activity of ITC toward dual-species biofilm of *S. aureus* and *S. uberis* at different concentrations at exposure time of 30 s **(B)**. The relative proportion of each bacterial component is expressed at the % of the total population in mixed biofilm. Mean % from tetrad coupons (+SD) are plotted.

The total colony-forming unit counts obtained from mono-species biofilms formed within 24 h on untreated coupons were 10^6^–10^7^ cfu ml^−1^ for all strains (Figure 3A). The anti-biofilm activity of ITC increased with increased concentration, eventually achieving the killing of *E. coli, S. aureus*, and MRSA mono biofilms below detection level with MBEC values of 125, 31.3, and 31.3 μg ml^−1^, respectively. ITC was the least effective toward *P. aeruginosa* biofilm, as the highest tested concentration (125 μg ml^−1^) was not sufficient for eradication, although it achieved a 5 log_10_ reduction in cell numbers. The individual contribution of *S. aureus* and *S. uberis* in the formation of dual-species biofilm was about 47 and 53%, respectively (Figure 3B). The treatment with a 15.6 μg ml^−1^ dose of ITC decreased the proportions of both bacterial species to negligible levels within 30 s. The MBEC against mixed biofilm was recorded at 250 μg ml^−1^.

**Figure 4:**
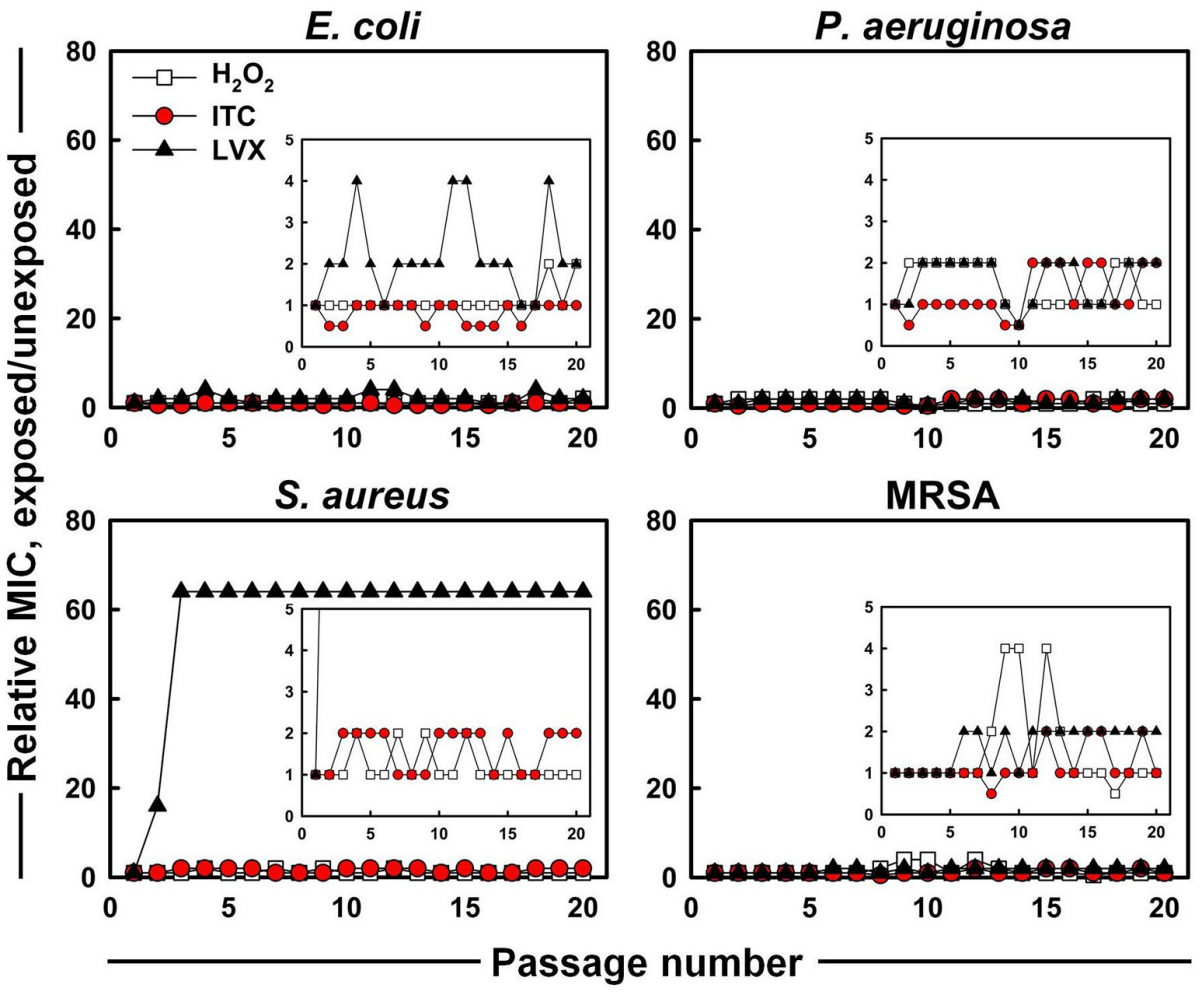
*****In vitro*** resistance acquisition of representative strains during 20 serial passaging in the presence of sub-inhibitory levels of H_**2**_O_**2**_, ITC and LVX tested by broth microdilution**. The relative MIC was the normalized ratio of the MIC obtained for the exposed subculture to the MIC that was obtained from unexposed culture; the insets show the expanded y-axis.

The evolution of MICs after successive exposures of *E. coli, P. aeruginosa, S. aureus*, and MRSA strains to sub-MIC concentrations of H_2_O_2_, ITC and LVX was assessed in this study (Figure 4). No significant increase and only 0.5 to 2-fold shifts of the ITC MICs were observed for all the test organisms. For H_2_O_2_, the largest increase in MIC was the 4-fold elevation toward MRSA, however, this change was reversed during subsequent passages. As for LVX, it showed maximum 4-fold increase in MIC toward *E. coli*, which was observed as a fluctuation rather than a permanent MIC rise. By contrast, *S. aureus* acquired resistance toward LVX. In the presence of sub-inhibitory concentrations of LVX, MIC of LVX rose 16-fold immediately after the first passage and arrived to 64-fold difference at passage three, which stayed stable over the next passages. On the other hand, LVX initially had a 64-fold higher MIC level (4 μg ml^−1^) toward MRSA compared to counterpart *S. aureus* strain (0.0625 μg ml^−1^), however it did not increase during *in vitro* evolution.

### Analysis for reactive species in the test antimicrobial mixtures

The antimicrobial solutions were analyzed using a number of analytical methods. The content analysis of 1% H_2_O_2_ and KI or/and KSCN combination mixtures is given in Table 2. The semi-quantitative test for peroxide presence showed that H_2_O_2_ was depleted in H_2_O_2_/KI and H_2_O_2_/KI/KSCN (ITC) mixtures, whereas H_2_O_2_/KSCN yet contained very small amount of unreacted peroxide. The hydroxyl radical content was the highest in H_2_O_2_/KI mixture, though it was also detected in the other combination mixtures. A high OSCN^−^/OI^−^ level was observed in H_2_O_2_/KSCN and H_2_O_2_/KI/KSCN solutions. Though the literature implies that OI^−^ (Bosch et al., 2000), as well as other hypohalous acids (Landino et al., 2008; Gau et al., 2015) can be measured by TNB-to-DTNB oxidation method we detected little amount of OI^−^ in the H_2_O_2_/KI mix. H_2_O_2_ itself is oxidizing the TNB (Landino et al., 2008) giving a false positive results for OSCN^−^/OI^−^ content consideration. However, most of the H_2_O_2_ in the mixtures were reacted and any oxidation of TNB in the mixtures was counted for OSCN^−^/OI^−^. Iodine, as it was expected, was detected in H_2_O_2_/KI, H_2_O_2_/KI/KSCN, PVP-I, and Lugol's mixtures. ITC contained considerable amount of molecular iodine, surpassing even PVP-I. Among all the test antimicrobials ITC was the one most possessing an assembly of reactive oxygen and iodine species in substantial quantities. The content analysis of ITC mixture on other occasions exhibited similar compositional profile.

**Table 2:** **Composition of 1% antimicrobial mixtures**.

**Antimicrobial solution**	**H_2_O_2_, +/−**	**·OH, mM**	**OSCN^−^/OI^−^, mM**	**I_2_, mM**
H_2_O_2_	+	0.14 ± 0.03	0.63 ± 0.03	−
H_2_O_2_/KI	−	1.04 ± 0.01	0.19 ± 0.02	0.95 ± 0.07
H_2_O_2_/KSCN	+	0.13 ± 0.04	1.24 ± 0.00	−
H_2_O_2_/KI/KSCN	−	0.19 ± 0.06	1.24 ± 0.00	9.7 ± 0.00
PVP-I	−	−	−	3.9 ± 0.14
Lugol	−	−	−	30.75 ± 0.35

*Data are given as mean values (± SD) of duplicates*.

**Figure 5:**
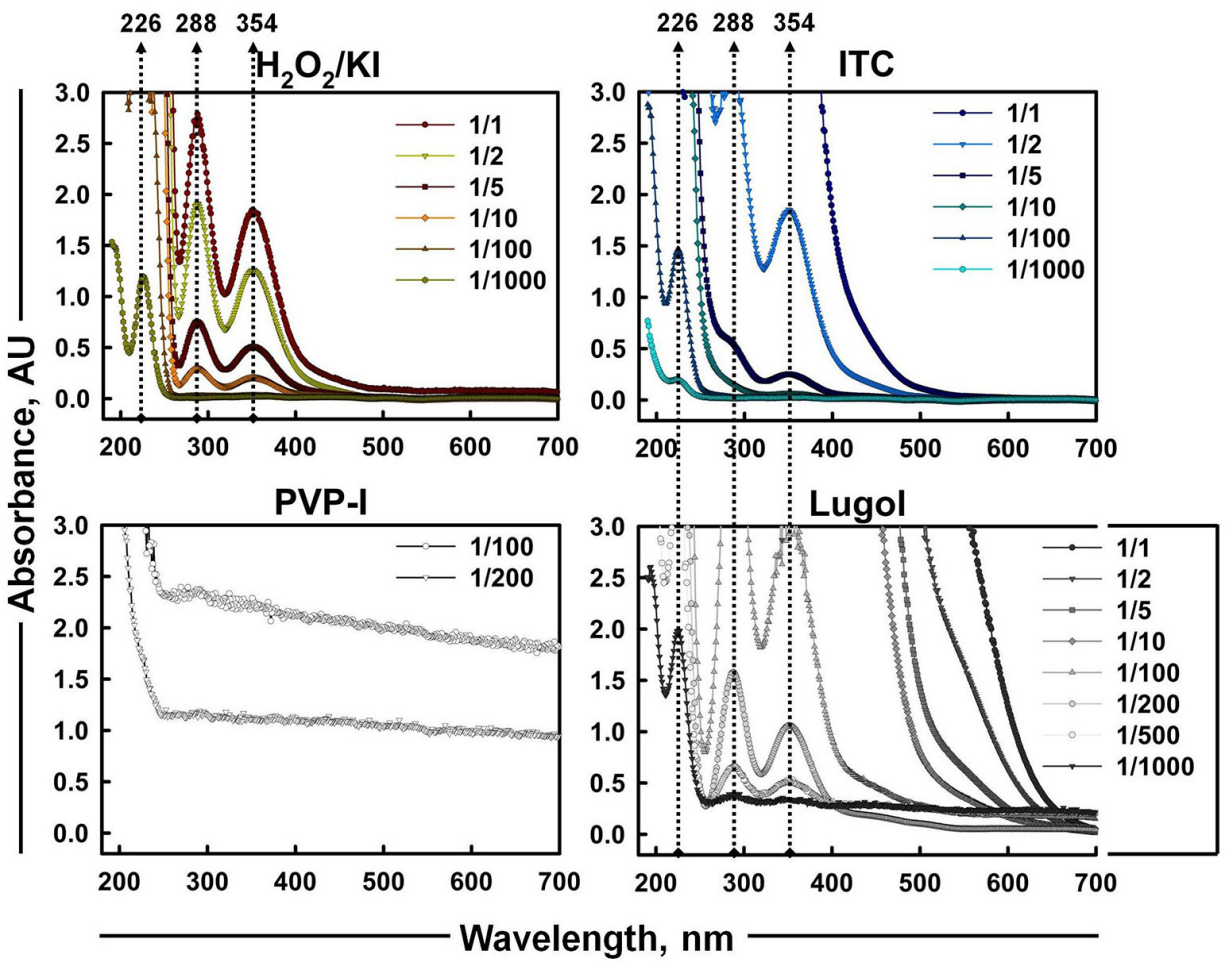
**UV-Vis absorption spectra of aqueous solutions of H_**2**_O_**2**_/KI, H_**2**_O_**2**_/KI/KSCN (ITC), PVP-I, and Lugol's iodine at different dilutions**. Absorption peaks of I^−^ (226 nm) and ${\text{I}}_{3}^{-}$ (288 and 354 nm) are shown with dotted arrowlines.

As iodine was believed to be the most considerable contributor of ITC antimicrobial action, the presence of different iodine species (I^−^, I_2_, and ${\text{I}}_{3}^{-}$) in iodine containing mixtures of H_2_O_2_/KI, H_2_O_2_/KI/KSCN, PVP-I, and Lugol was also visualized by UV-Vis spectrometry (Figure 5). Scanning of different dilutions of 1% ITC in the range of 190–700 nm wavelengths showed that 1/2 and 1/5 diluted solutions had two peaks at about 288 and 354 nm, which are known to be bands of tri-iodide ions (Gazda et al., 2004; Wei et al., 2005; Mazumdar et al., 2010; Mertes et al., 2012; Kireev and Shnyrev, 2015). However, characteristic bands of I_2_ at about 203 (Wei et al., 2005) and 460 nm (Wei et al., 2005; Kireev and Shnyrev, 2015) were not distinguishable in all the measured dilutions of ITC. Gazda et al. (2004) and Punyani et al. (2007) indicated that the peak at 460 nm had a very low energy, whereas, Kireev and Shnyrev (2015) described I_2_ peaks at 203 and 460 nm as very large bands, consequently, I_2_ peaks could have been hidden because of spectral interference or of weak intensity. Moreover, at 1/1,000 dilution of ITC we could detect a band applying to iodide ion with absorption maximum of 226 nm. This suggested that KI was not completely depleted in H_2_O_2_/KI/KSCN system and iodine present in the solution could occur in ${\text{I}}_{3}^{-}$ form. H_2_O_2_/KI mixture showed two peaks of ${\text{I}}_{3}^{-}$ at 1/1, 2, 5, 10 dilutions and only I^−^ peak at 1/1,000 dilution. Similarly, Lugol displayed two ${\text{I}}_{3}^{-}$ bands at 1/100, 200, 500 dilutions, I^−^ peak at 1/1,000 dilution, and none of I_2_ characteristic bands at 460 or 203 nm. In contrast to published evidence (Oster and Immergut, 1954; Mazumdar et al., 2010), we could not acquire iodine bands from PVP-I solution, possibly, because of a spectral interference from PVP polymer.

### Effect of ITC on cellular components

**Figure 6:**
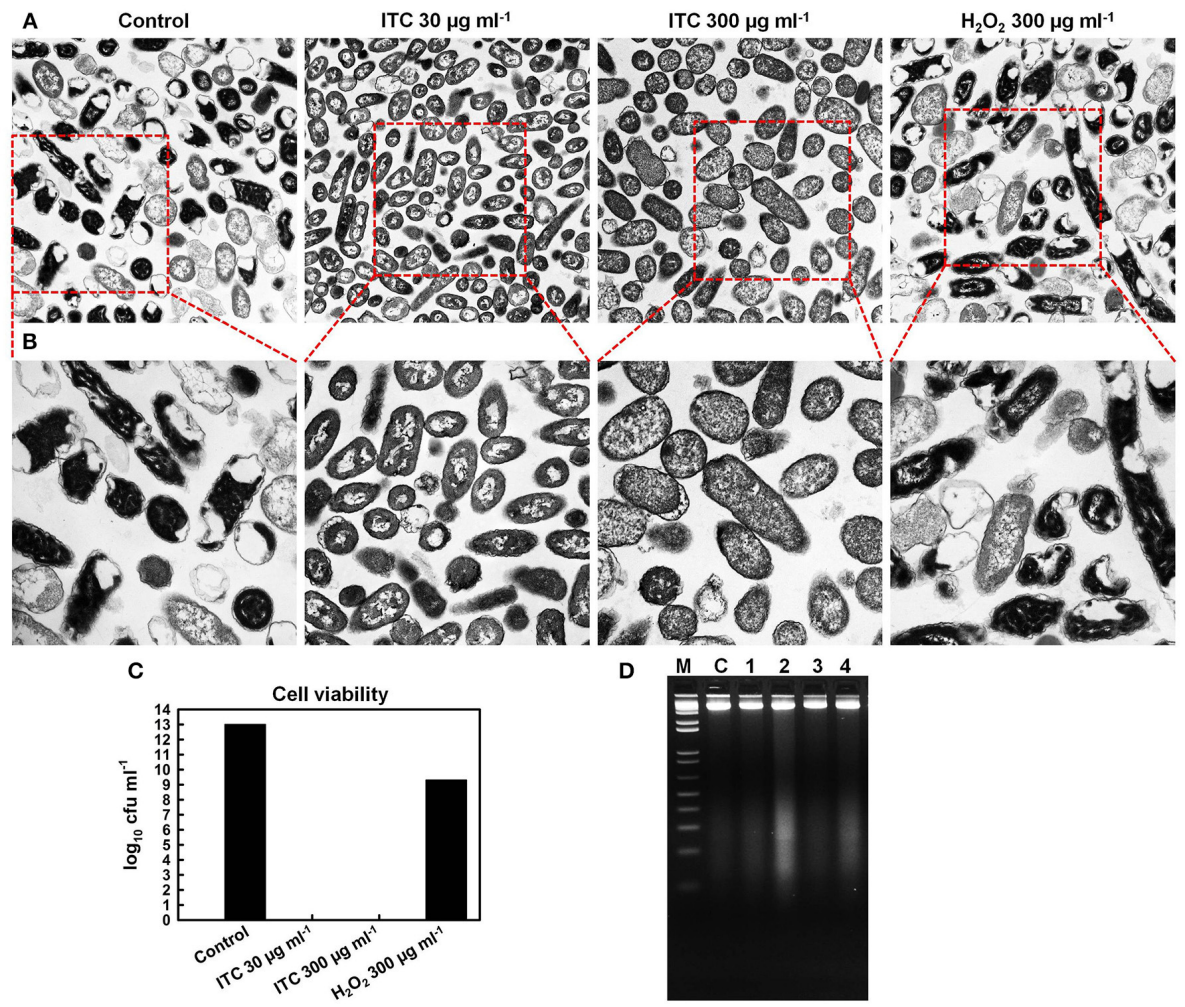
**Effect of ITC on cellular components of ***E. coli*****. Transmission electron micrographs of untreated control, treated with ITC at 30 and 300 μg ml^−1^, and H_2_O_2_ at 300 μg ml^−1^ for 2 h, and visualized at 10,000× **(A)** and at 20,000× magnifications **(B)**. Cell counts (log_10_ cfu ml^−1^) of viable *E. coli* cells from the same samples prior to preparation for TEM **(C)**. Bacterial DNA damage detection by agarose gel electrophoresis **(D)**. Samples 1, 2, 3, and 4 *E. coli* cells were treated 30 min with ITC at 30 and 300 μg ml^−1^, H_2_O_2_ at 30 and 300 μg ml^−1^, respectively. gDNA was extracted and gel electrophoresis was performed for examination of DNA cleavage (DNA smear). C is gDNA from untreated cells, M is a 1 kb DNA marker.

TEM was used for direct observation of ITC-induced ultrastructural alterations in *E. coli*. Visualization of the micrographs revealed that the untreated control specimen contained more cells with electron-dense (appears dark) cytoplasm, rich in ribosomes (dark granules), which form a riboplasm (Figures 6A,B). The DNA (electron-light material) of the untreated cells was distributed randomly within the riboplasm. While *E. coli* cells in ITC 30 μg ml^−1^ treated group appeared to have more internal translucent areas—ribosomal grains looked light-colored and DNA appeared as swirls in the middle of the cells, however, cells looked intact. Cells in ITC 300 μg ml^−1^ treated sample appeared to have more dissolved picture—DNA appeared to be fragmented and ribosomal grains were more scarcely distributed, but then again cells were intact. In addition, the image of H_2_O_2_ 300 μg ml^−1^ treated group resembled the scene from the control sample, composed of mostly cells with “dark matter.” Whilst, viable cell counts showed complete eradication of 10^13^ cfu ml^−1^ bacterial load with ITC at high, and even low concentrations within 2 h exposure (Figure 6C). H_2_O_2_ at 300 μg ml^−1^ concentration resulted only in 4 log_10_ reduction of *E. coli*.

To confirm the observations from TEM microscopy and to show DNA cleavage in bacterial cells, *E. coli* cells were treated with low (30 μg ml^−1^) and high (300 μg ml^−1^) concentrations of ITC and H_2_O_2_, followed by extraction of gDNA and agarose gel electrophoresis. DNA smears were detected in *E. coli* cells treated with ITC and H_2_O_2_ at their highest tested concentrations—300 μg ml^−1^ (Figure 6D, samples 2 and 4, respectively).

**Figure 7:**
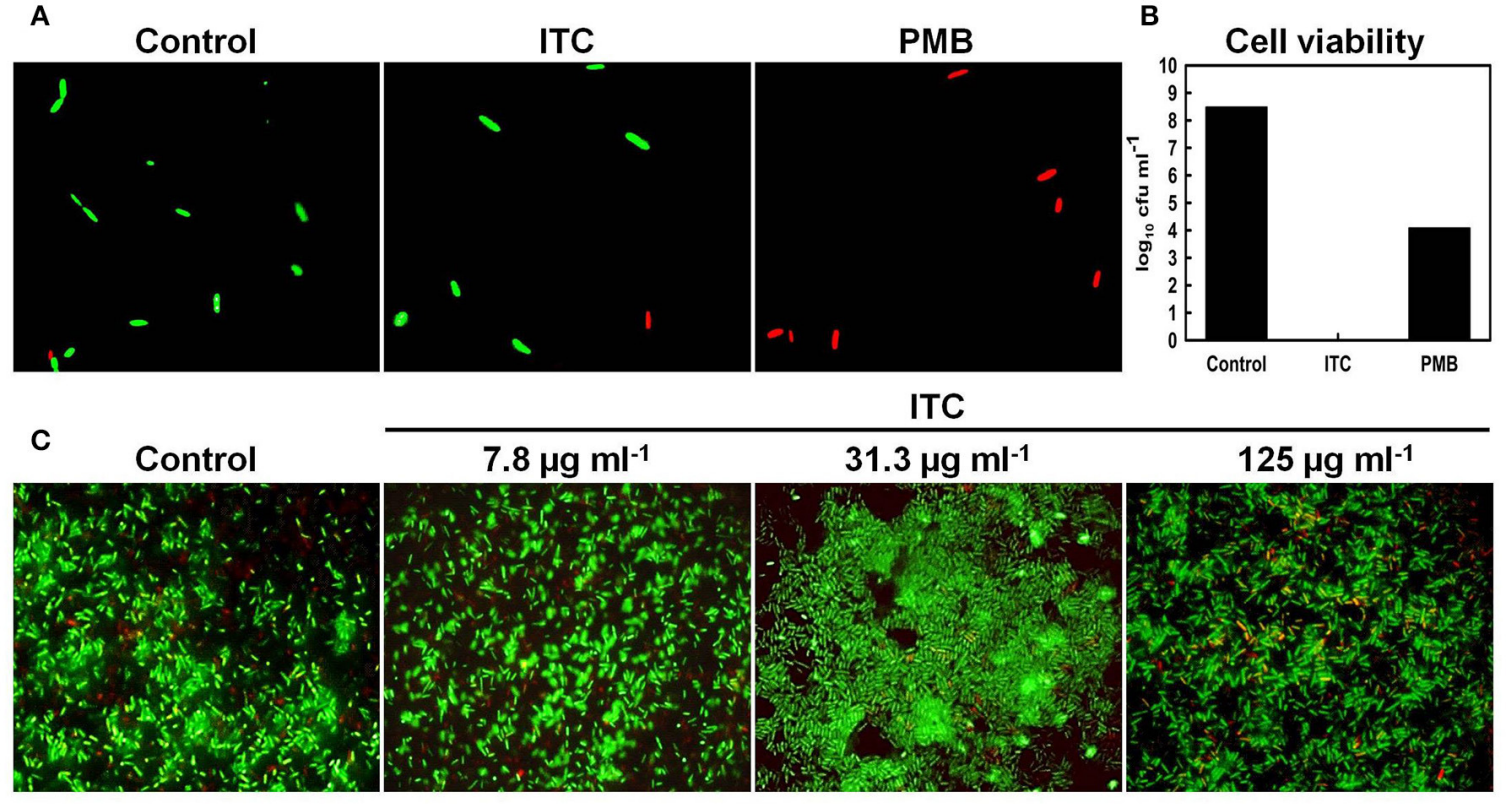
**Influence of ITC on ***E. coli*** membrane integrity assayed by live/dead staining**. Fluorescence micrographs of *E. coli* planktonic cells in exponential phase treated with ITC at 125 μg ml^−1^ and polymyxyn B at 1 μg ml^−1^ upon 1 h and stained with LIVE/DEAD BacLight stain **(A)**. Cell counts (log_10_ cfu ml^−1^) of viable *E. coli* planktonic cells prior to live/dead staining **(B)**. Representative images of *E. coli* biofilm grown on glass microscope slides upon 24 h, than treated 10 min with different concentrations of ITC and stained with LIVE/DEAD BacLight stain **(C)**. Membrane is expected to be intact in green-fluorescent and compromised in red- fluorescent cells.

To further support TEM results and to exhibit that ITC was not compromising membrane integrity of *E. coli*, live/dead co-staining technique was used. It is a two-color fluorescence assay based on membrane integrity that simultaneously determines live and dead cells. The membrane-permeable SYTO 9 (green-fluorescent nucleic acid stain) labels all bacteria in a population (intact and ruptured), in contrast, propidium iodide (red-fluorescent nucleic acid stain) penetrates only bacteria with damaged membranes, causing a reduction in the SYTO 9 stain fluorescence when both dyes are present (Stocks, 2004). Fluorescent micrographs of live/dead stained *E. coli* planktonic cells showed no difference between ITC-treated and untreated control groups, displaying mostly green stained cells (Figure 7A), yet no viable cells were recovered on agar plates from ITC-treated cultures (Figure 7B). Whereas, red control PMB-treated group displayed mostly red cells and a 4 log_10_ reduction in cfu numbers when exposed to 2 × MIC (Figures 7A,B). In addition, *E. coli* biofilm cells treated with different concentrations of ITC showed predominantly green-fluorescent cells, similar to the untreated control, with some red-fluorescent cells in the aggregates (Figure 7C).

## Discussion

Multicellular organisms have developed different enzyme systems, which offer antimicrobial activity and play a significant role in the defense of the host organism. Approaches simulating the natural antimicrobial systems have already found application in many fields and further development of the research field is both promising and necessary, given the global antibiotic crisis. We have developed an easy-to-prepare antimicrobial composition ITC, inspired by naturally occurring peroxidase systems, but with distinctive antimicrobial activity. A biocidal composite is formed from the combination of hydrogen peroxide, iodide and thiocyanate salts at a ratio of 1:1:1 (v/v/v). Incorporation of I^−^ and SCN^−^ into peroxidase systems have been poorly investigated and contradictory results have been reported about the antimicrobial action of this combination; some are supporters of synergistic, the others, antagonistic interactions. For example, Ihalin and group successively reported that the addition of SCN^−^ into the peroxidase/H_2_O_2_/I^−^ system abolished the bactericidal activity of the oxidized halide (Ihalin et al., 1998, 2001). Similarly, Ahariz and Courtois (2010) demonstrated a competition between I^−^ and SCN^−^, reporting that the addition of SCN^−^ to peroxidase/G/GOD/I^−^ system (G/GOD: glucose/glucose oxidase as source of hydrogen peroxide) decreased the antifungal effect of the system.

On the contrary, Galley et al. (1997) suggested the incorporation of I^−^ in their commercial peroxidase/SCN^−^/G/GOD preservative system, aiming to extend the spectrum of activity of the system—OI^−^ being much more effective against yeast and molds than OSCN^−^, and the latter efficient against bacteria. Likewise, Bosch et al. (2000) confirmed the synergistic effect between I^−^ and SCN^−^.

However, all these reported antimicrobial systems involved a peroxidase enzyme and there could be a competition between two substrates for the oxidation by the enzyme. Whereas, in our system it is H_2_O_2_ that is oxidizing both substrates, generating a “cocktail” of various halogenating species. The potent antimicrobial activity of ITC mixture is not solely attributed to the presence of one, but rather to the combination of different antimicrobial species, nominating it for an attractive weapon to kill pathogenic microorganisms.

Here, we examined the synergism of antimicrobial activity between a mixture of H_2_O_2_ with two substrates (I^−^ and SCN^−^) against representative Gram^−^ and Gram^+^ organisms. The disc diffusion and broth microdilution methods used in our study suggested a putative cooperation between two substrates. Specifically, the observations with these two methods indicated synergistic antimicrobial action against two strains tested—*E. coli* and *P. aeruginosa*, whereas, comparable effects toward *S. aureus* and MRSA (Figures 1A,B, Table 1). One of the features of ITC was that it was the most rapidly bactericidal among the antimicrobials surveyed. ITC killed bacteria very rapidly, and did not just arrest their growth (Figure 2). Whereas, other test antimicrobials showed slow killing kinetics or were bacteriostatic. Since the H_2_O_2_/KI/KSCN triple combination is successfully eliminating pathogenic bacteria and what is more, causing the death of bacteria, it could be considered as a potential antimicrobial for treatment of bacterial infections. These observations will lead us to further investigate the potential of ITC on an extended list of antibiotic resistant clinical isolates.

Microorganisms commonly attach to organic or inorganic surfaces and exist in a biofilm mode. Biofilms are ubiquitous and can occur on a wide variety of surfaces, including living tissues, indwelling medical devices, industrial or potable water system piping, natural aquatic systems, household surfaces, etc. (Donlan, 2002). Biofilms are responsible for a number of infectious diseases. Cystic fibrosis, native valve endocarditis, otitis media, and chronic prostatitis all emerged from biofilm-associated microorganisms (Donlan and Costerton, 2002). Microbial biofilms developed on or within medical indwelling devices play a central role in pathogenesis of patient infection (Trautner and Darouiche, 2004). Oral biofilms can form a dental plaque on the surfaces of teeth. Further development of dental plaque can lead to serious complications, such as caries, gingivitis, and periodontitis (Hope and Wilson, 2004). Furthermore, bacterial biofilms play a significant role in chronic wound infections and are major barrier to wound healing (Phillips et al., 2010). While biofilm development is not a prerequisite for persistent infection, eradication of biofilm-based infections is particularly difficult (Archer et al., 2011). It is commonly accepted that biofilms are less susceptible toward antimicrobial treatment than planktonic cells, and many antimicrobial agents show reduced efficacy in eliminating biofilms. Our study showed that H_2_O_2_/I^−^/SCN^−^ mixture was able to effectively eradicate mono-species biofilm mode of Gram^−^ and Gram^+^ pathogenic strains within a short 10 min exposure (Figure 3A). Though biofilms can be formed by a single bacterial species, more commonly biofilms represent complex mixed communities (Elias and Banin, 2012). Studies showed that multi-species biofilms tend to withstand antimicrobial treatment or disinfection more efficiently (Leriche et al., 2003; Burmølle et al., 2006; Kara et al., 2006). In our study, however, ITC could readily eliminate even established dual-species biofilm community within just 30 s (Figure 3B). The treatment of dual-species biofilm for 10 min at the tested concentrations (15.6–250 μg ml^−1^) resulted in no culturable cells (data not shown), thus, the effect of 30 s contact time is presented. Oppositely, the treatment of *P. aeruginosa* mono-species biofilm over 10 min resulted in viable counts, thus, the effect of 30 s on mono-species biofilm was not assessed. Anyhow, relatively short contact times and low concentrations were required to eliminate mono- and dual-species biofilms. We shall also note here that biofilms were established over recirculating batch culture, thus, considering nutrient limitation and bacterial waste accumulation, they were grown for 24 h. Since biofilms tend to become less susceptible as they age, testing the antimicrobial efficacy of ITC on 1-day old biofilms may be a limitation, and future research can potentially address it. In any case, the novel antimicrobial complex could be considered a useful approach in the fields of medical, food, and water microbiology to prevent and control biofilm-associated contaminations.

Successful inhibition of potential pathogenic bacteria would allow possible use of the new iodo-thiocyanate system as a potential therapeutic or disinfectant. The possibility of the development of resistance to an antimicrobial therapeutic is an important factor in determining its potential. Therefore, ITC was tested against four test organisms to assess whether emerging resistance could be developed using multiple exposures to ITC. *E. coli, P. aeruginosa, S. aureus*, and MRSA were cultured for 20 consecutive passages in the presence of ITC, and compared with LVX (Figure 4). No increase was recorded for ITC MIC toward all the test organisms, whereas, LVX MIC increased 64-fold for *S. aureus*. These results imply that ITC is unlikely to introduce a resistance in bacteria typically observed with antibiotic usage. This is probably due to a multiplicity of targets, requiring simultaneous mutations within the organism. Similar cases of “resistance resistant” antimicrobial approaches are photodynamic therapy and cold atmospheric plasma that avoid triggering resistance emergence by hitting multiple targets simultaneously (Tavares et al., 2010; Vatansever et al., 2013; Matthes et al., 2014).

While the study presented here would suggest that ITC could be considered as a biocidal agent suitable for disinfection applications, its use in infection treatment will of course be based on whether it can be used without serious harm to the human or animal. The cytotoxic aspects of ITC should thus be evaluated in future research.

Analysis of the chemical composition of the ITC mixture revealed that a bactericidal property of it is due to coupling chemistry of various cidal species. The reactive mixture contained hydroxyl radical, hypo(pseudo)halite ions, and iodine, the latter being the dominant antimicrobial component (Table 2). Iodine in the form of tri-iodide ions were detected also by UV-Vis spectrometry (Figure 5). Individually, these species found in ITC mixture possess antimicrobial properties and target different sites in microorganisms. The hydroxyl radical can directly oxidize all biomolecules, however, the DNA is an especially favored target of it (Farr and Kogoma, 1991; Imlay, 2003). Biological activities of oxidized SCN^−^ and I^−^ are summarized in the review of Bafort et al. (2014). The review highlights that OSCN^−^ targets sulfhydryl group of peptides and proteins, inhibiting glycolysis, respiration and glucose transport in bacteria. However, not all thiols are sensitive toward OSCN^−^, so the reversible inhibition is occurred, indicating the bacteriostatic effect of OSCN^−^. Products of oxidized iodide (OI^−^/I_2_) can affect thiol groups, NAD(P)H, and thioether groups, inhibiting bacterial glycolysis, respiration, glucose transport and pentose phosphate pathways. In contrast, the oxidized I^−^ species are reactive toward all thiol groups coming up as bactericidal. Moreover, Thomas and Aune (1977) reported that oxidation of cell components with I_2_ yielded in the reduction of I_2_ back to I^−^, so that as a result I^−^ was not consumed. Released I^−^ could be reoxidized and take part again in the oxidation of other protein sulfhydryls. Therefore, one iodide ion could oxidize many cell components. In any case, although, reactive oxygen and iodine species were present in the mixture of ITC, the full chemistry and other possible reactive species generated therein are yet to be clarified.

TEM was used in order to gain an insight on the bactericidal effect of ITC. Similarly, Schreier et al. (1997) carried out TEM study to elucidate the molecular effect of PVP-I on *E. coli, S. aureus*, and *Candida albicans* cell ultrastructures. They recorded rapid partitioning of riboplasm and pronounced coagulation of nuclear material without visible cell rupture or lysis in *E. coli* and *S. aureus*. Under our observations, significant morphological changes occurred in *E. coli* cells subjected to ITC treatment (Figures 6A,B). Cells treated with low concentration (30 μg ml^−1^) of ITC were more translucent in peripheral ribosome-rich cytoplasmic region and contained light-colored ribosomal grains, as compared to the untreated control. ITC may have bound with ribosomal protein thiol groups and “loosened” ribosomes. These groups are involved in molecular interactions that maintain the integrity of the ribosomes, keeping them in a compact configuration (Beeley, 1970). In addition, DNA swirls emerged as a notable bright electron zones in the center of the cells, suggesting that ITC could have an effect on DNA condensation (Figures 6A,B). Similar DNA swirling and condensation was observed in *E. coli* and *S. aureus* cells treated with silver ions claiming that Ag^+^ treated cells lost their replication ability (Feng et al., 2000). Increase in the concentration of ITC (300 μg ml^−1^) created even more translucent cytoplasmic region and scarcely distributed ribosomal granules (Figures 6A,B). The increase of ITC concentration in the cells also brought to disappearance of distinctive central nuclear zone, indicating the fragmentation of DNA swirls. Likewise, DNA cleavage was observed in DNA gel electrophoresis study—ITC was inducing DNA smear when cells were treated at 300 μg ml^−1^ concentration (Figure 6D, sample 2). However, at both concentrations the cells exhibited notable alterations in the cell cytoplasm without losing the cell membrane integrity (Figures 6A,B). Live/dead staining assay supported these observations that ITC was not compromising membrane integrity in *E. coli* (Figures 7A,C). We should note that at high (300 μg ml^−1^) and even low (30 μg ml^−1^) concentrations ITC was totally eliminating bacteria within 2 h of exposure (Figure 6C), meaning that observable morphological changes should be the representatives of its bactericidal action. The above discussion led to the suggestion that the antimicrobial effect of ITC is perhaps a result of simultaneous events.

Herein, we report on an antimicrobial composition, which exhibits a broad-spectrum activity against pathogenic bacteria in planktonic and biofilm forms, rapidly eliminating them. Moreover, our *in vitro* study showed that the novel antimicrobial does not promote the acquisition of resistance in bacteria. Possession of low MIC, rapid bactericidal activity and little possibility for triggering resistance emergence are positive characteristics for potential therapeutics or biocides. Therefore, it is anticipated that ITC might in future find use as a novel antimicrobial agent, to treat infections and/or to decontaminate surfaces. However, further biocompatibility studies will be necessary to establish its safety as an antimicrobial agent.

## Author contributions

LT designed and completed the experiments, wrote and reviewed the manuscript. PM, RF, and VO contributed to the conception and design of the experimental work and the interpretation of the results. VO, RF, and GF critically reviewed and edited the manuscript.

## Funding

This work was funded by the Irish Research Council Government of Ireland Postgraduate Scholarship Scheme (GOIPG/2013/584) and by Enterprise Ireland (CF/2011/1605).

### Conflict of interest statement

The antimicrobial described in this manuscript is subject to a patent application (application number EP2987408A1). Any Intellectual Property arising shall be owned by the National University of Ireland Galway. The authors declare that the research was conducted in the absence of any commercial or financial relationships that could be construed as a potential conflict of interest.

## Abbreviations

ITC: iodo-thiocyanate complex
MPO: myeloperoxidase
LPO: lactoperoxidase
SPO: salivary peroxidase
PMB: polymyxin B
LVX: levofloxacin
PVP-I: povidone iodine
MRSA: methicillin-resistant *Staphylococcus aureus*
LB: lysogeny broth
LA: Lennox agar
PBS: phosphate-buffered saline
OD: optical density
cfu: colony-forming units
ZOI: zone of inhibition
MIC: minimum inhibitory concentration
MBC: minimum bactericidal concentration
MBEC: minimum biofilm eradication concentration
MRD: modified Robbins device
DMSO: dimethyl sulfoxide
MSA: methanesulfinic acid
DTNB: 5,5′-dithiobis(2-nitrobenzoic acid)
TNB: 5-thio-2-nitrobenzonic acid
TEM: transmission electron microscopy.
