# Supplementary figures and images for: Antibacterial Potential of an Antimicrobial Agent Inspired by Peroxidase-Catalyzed Systems
Source: Front Microbiol. 2017 May 2;8:680. doi: 10.3389/fmicb.2017.00680 (PMC5412088; doi:10.3389/fmicb.2017.00680)

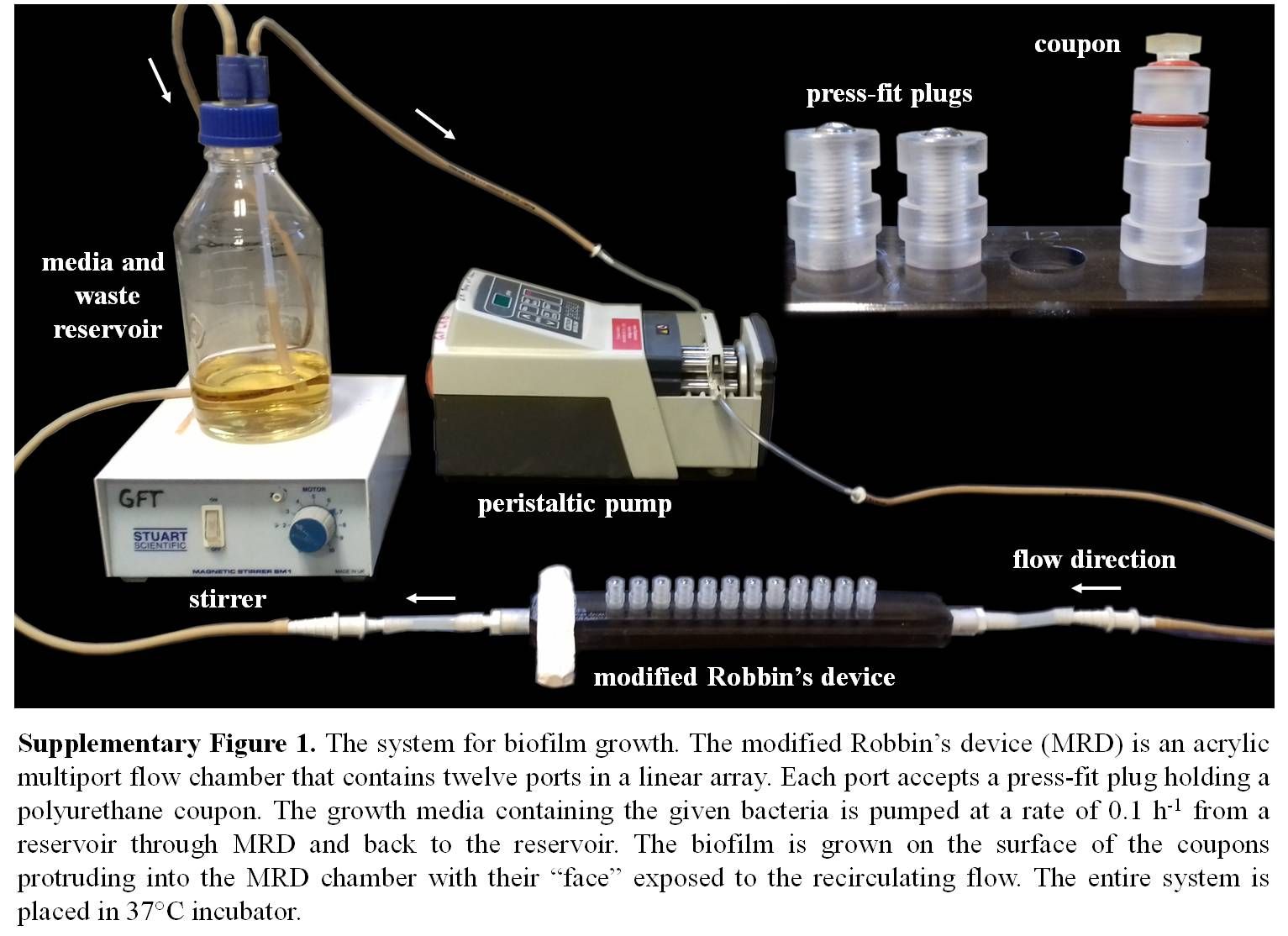

Supplement: Supplementary file 1 [file Image1.JPEG]
